# Sequential multimodal endoscopic therapy for recurrent extensive superficial basaloid esophageal squamous cell carcinoma with a 24-month follow-up

**DOI:** 10.1055/a-2944-3627

**Published:** 2026-09-08

**Authors:** Jing Huang, Wenconghui Wu, Xiaodan Zhang, Ying Chang

**Affiliations:** 1 Department of Gastroenterology 89674 Zhongnan Hospital of Wuhan UniversityWuhanHubeiChina

Superficial basaloid esophageal squamous cell carcinoma (SBSCC) is a rare and aggressive variant of squamous cell carcinoma characterized by high proliferative activity, atypical endoscopic appearance, and elevated recurrence risk. 1​ 2​ ​3​ Cases with extensive multifocal disease and recurrent behavior are clinically rare and lack standardized surveillance or treatment protocols. We report a case of extensive multifocal SBSCC with recurrence successfully managed by multimodal endoscopic therapy and intensive surveillance, achieving esophageal organ preservation.

**Fig. 1:**
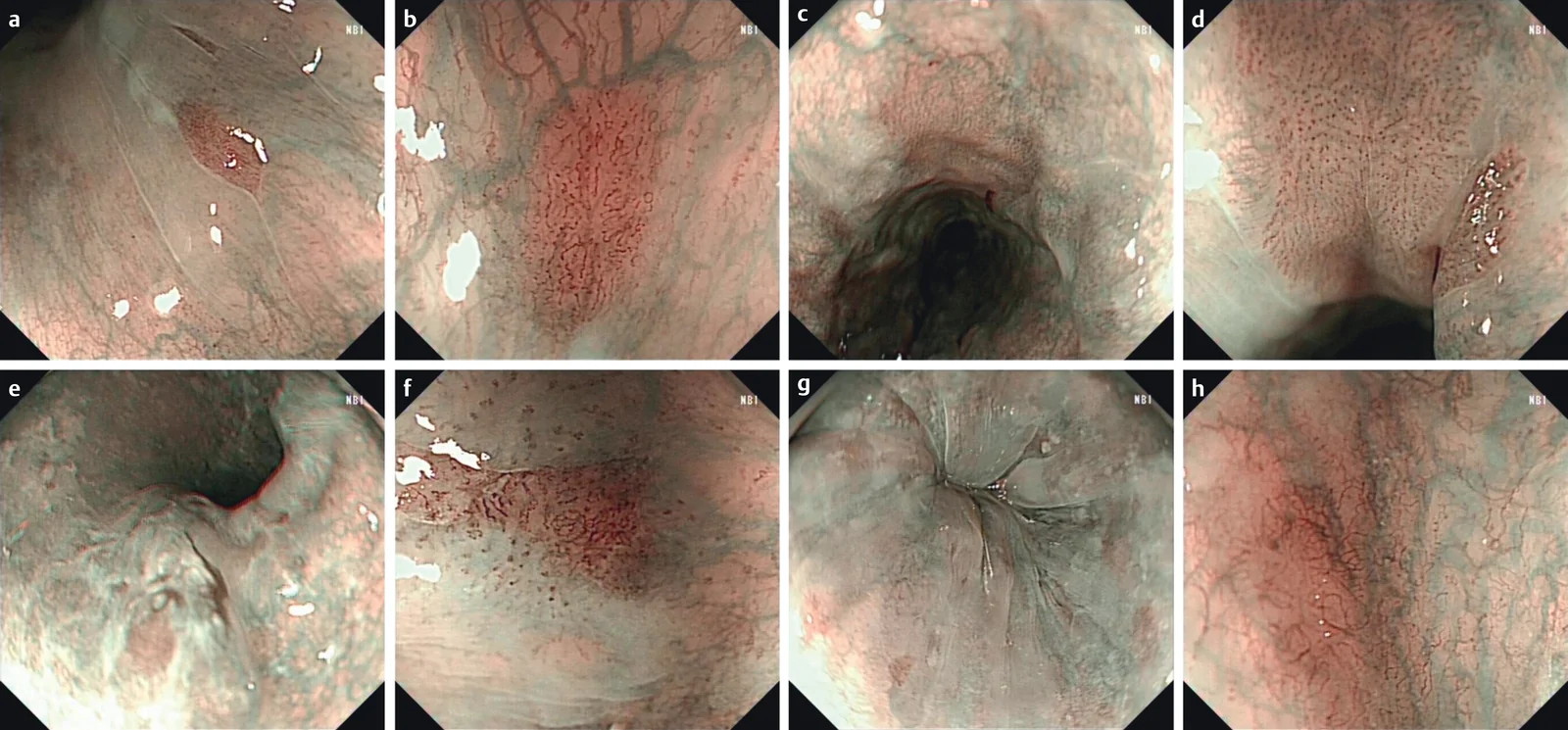
ME-NBI of four lesions (27, 30–32, 35, and 40 cm) show type R IPCLs. ( **a** , **c** , **e** , and **g** ) NBI and ( **b** , **d** , **f** , and **h** ) ME.

**Fig. 2:**
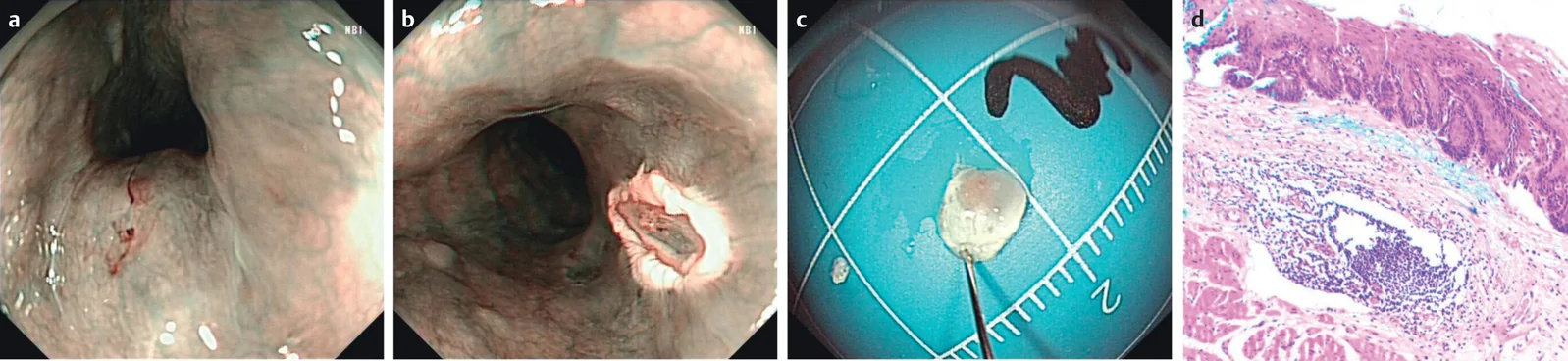
EMR for targeted biopsies. ( **a** ) Pre-EMR lesion. ( **b** ) Post-EMR defect. ( **c** ) Representative specimen (27 cm). ( **d** ) Histopathology: M2 SBSCC (27 cm, similar at 30–32, 35, and 40 cm).

A 61-year-old man presented with scattered type 0-IIb brownish lesions from 20 to 40 cm on initial screening endoscopy. Magnification endoscopy with narrow-band imaging revealed type R (reticular) intrapapillary capillary loops according to the Arima classification ( Fig. 1 ). Multiple targeted biopsies confirmed SBSCC with invasion limited to the lamina propria mucosa (M2). Lugol chromoendoscopy showed widespread Lugol-voiding lesions from 20 to 40 cm, confirming field cancerization ( Fig. 2 ). Given the patient’s strong desire for esophageal preservation, an individualized plan was developed.

**Fig. 3:**
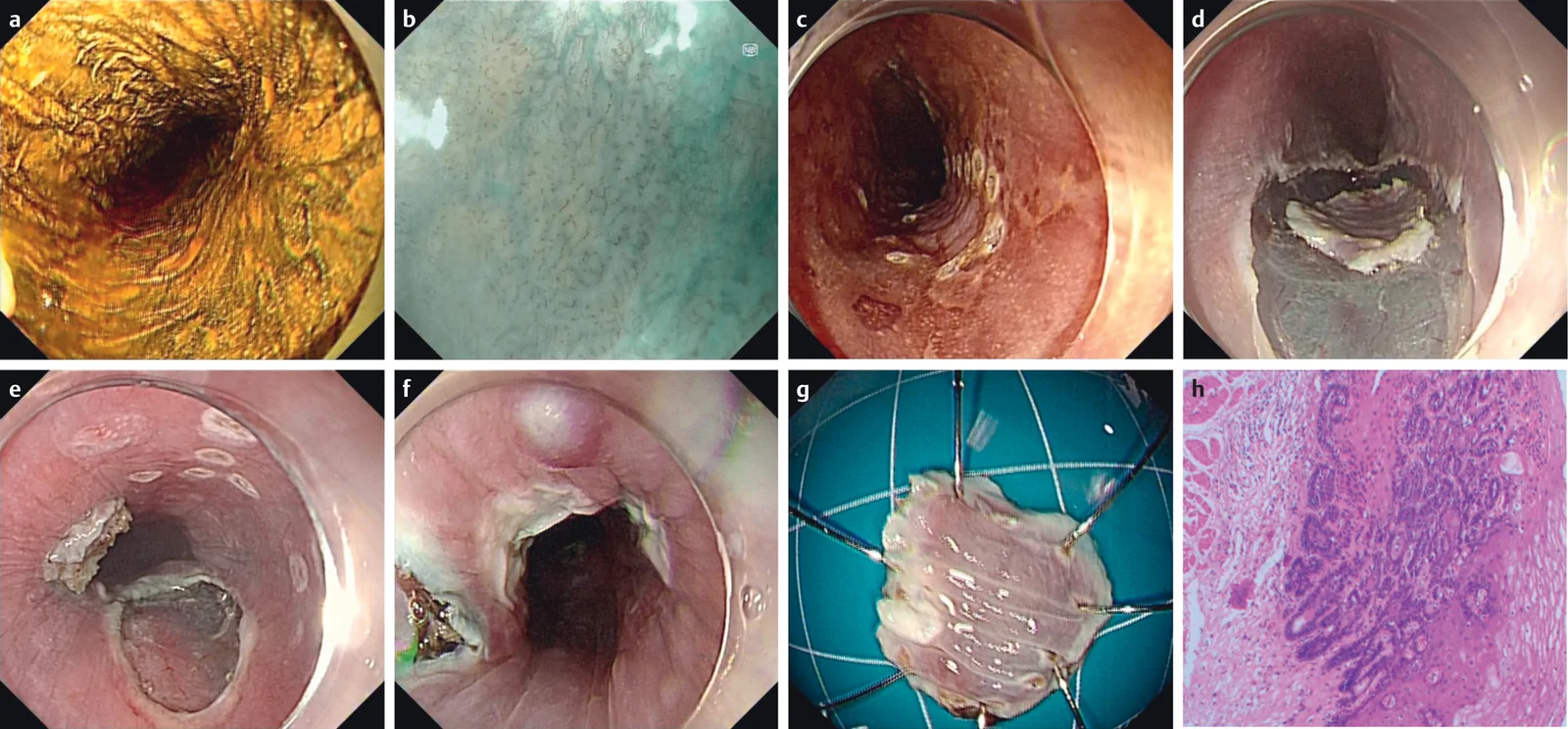
ESD. ( **a** ) Lugol (widespread LVLs). ( **b** ) ME-NBI (type R). ( **c–f** ) Marking, dissection, coagulation, and post-ESD defect. ( **g** ) Representative specimen (30–32 cm). ( **h** ) M2 SBSCC (20, 26, 27, 30, and 30–32 cm).

The initial treatment adopted a stratified intervention strategy: endoscopic submucosal dissection was performed on five major lesions (20, 26, 27, 30, and 30–32 cm); remaining scattered small lesions were precisely electrocoagulated. Postoperative pathology confirmed that all lesions were SBSCC (M2), consistent with the preoperative diagnosis ( Fig. 3 , Video 1 ).

**Fig. 4:**
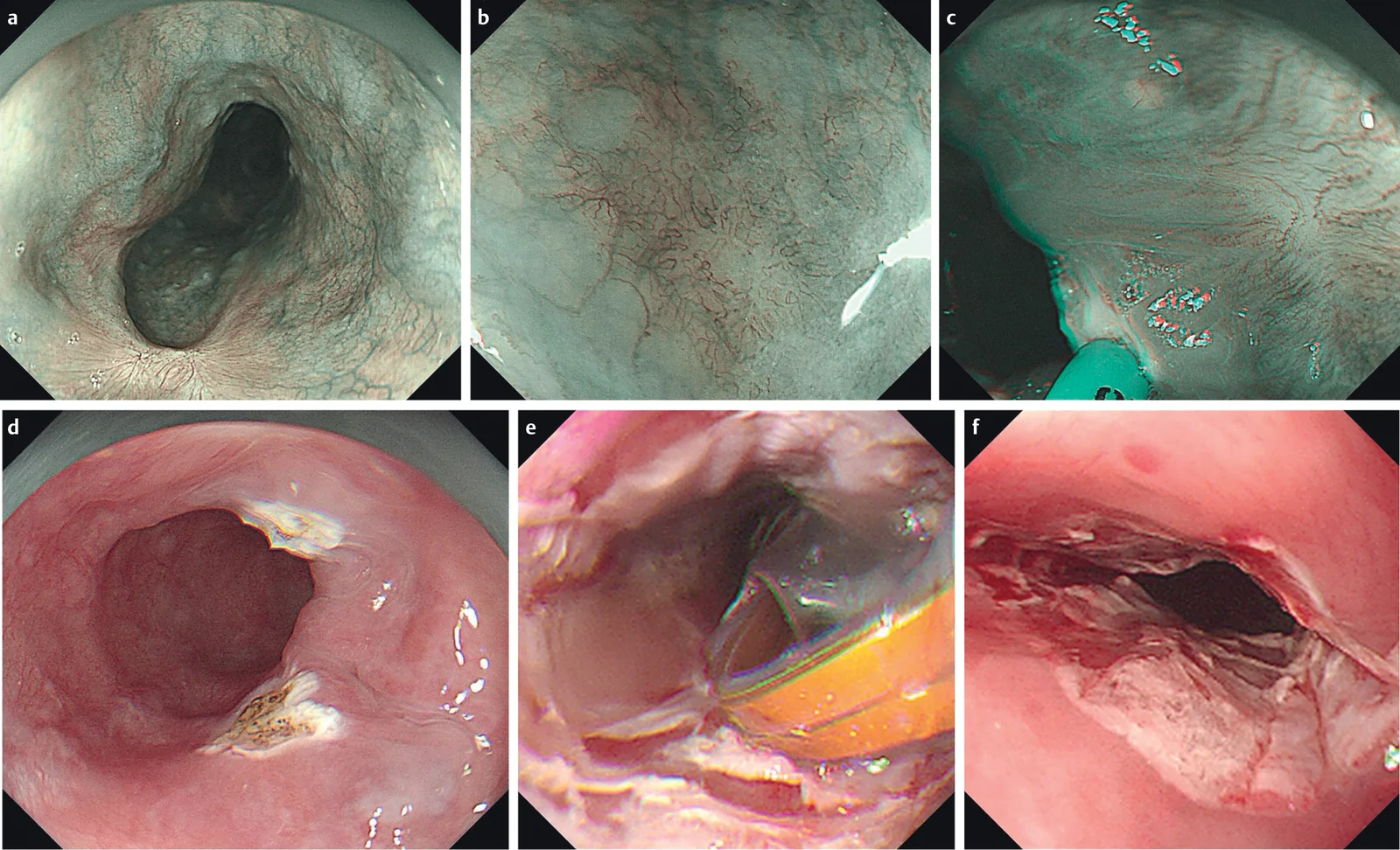
RFA with concurrent APC. ( **a** ) Background NBI. ( **b** ) ME (type R). ( **c** ) APC. ( **d** ) Post-APC mucosa. ( **e** ) A balloon-type RFA catheter. ( **f** ) Post-RFA appearance.

**Video 1** Sequential multimodal therapy and intensive surveillance for recurrent extensive SBSCC with a 24-month follow-up. Video URI:


At 4-month surveillance, multiple new small brownish lesions with type R appeared, confirming persistent new development and recurrent metachronous lesions. We used argon plasma coagulation (APC) for visible small lesions (<5 mm) and applied balloon-type circumferential radiofrequency ablation to the background mucosa at 30–40 cm to reduce metachronous recurrence. 4​ Six segments were treated with two 10 J/cm ^2^ ablations per segment and a 0.5 cm overlap between adjacent segments, achieving complete coverage of the high-risk area ( Fig. 4 , Video 1 ).

**Fig. 5:**
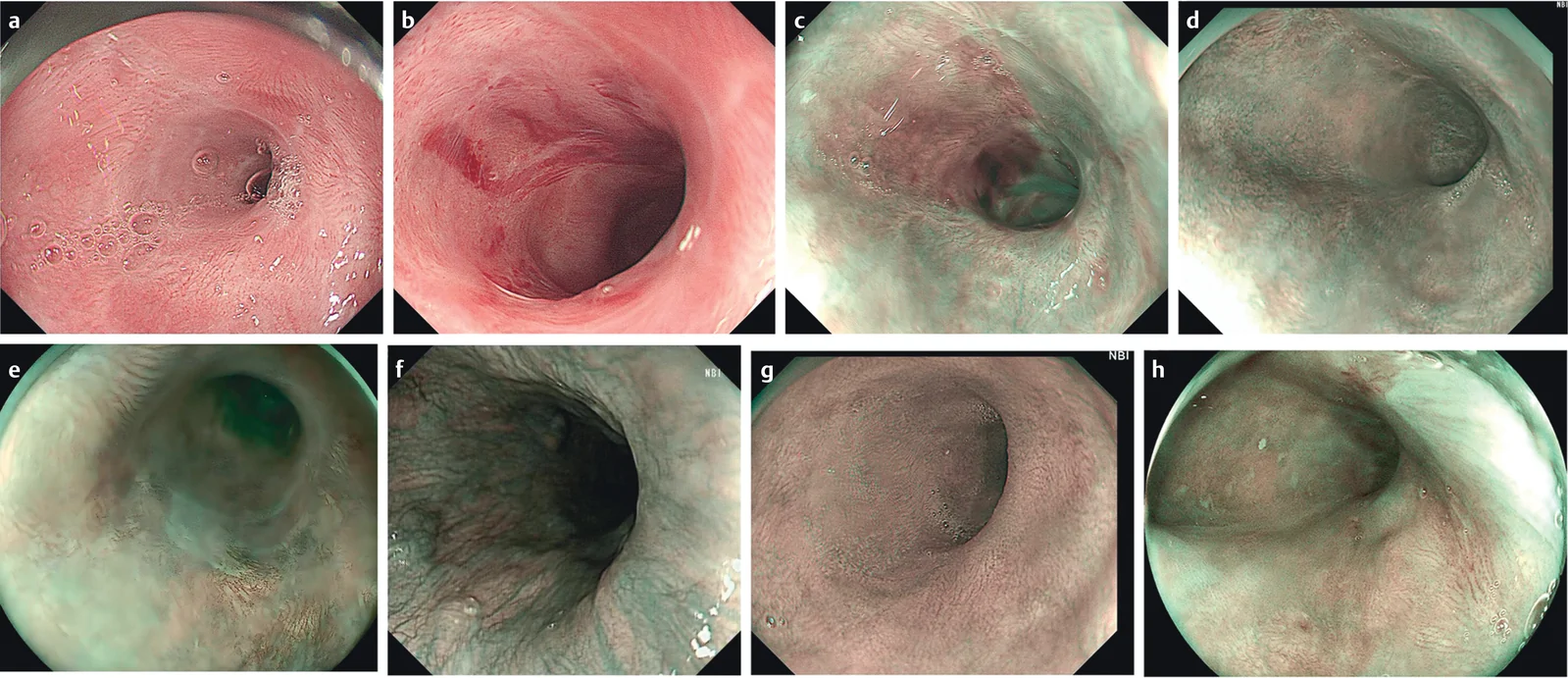
Follow-up. ( **a** ) Mild stricture at 1 month. ( **b** ) After forced scope passage. ( **c–h** ) NBI at 1, 4, 8, 13, 18, and 24 months.

Intensive surveillance was performed every 4 to 6 months, with APC ablation for suspicious lesions. A mild stricture at 1 month resolved after forced passage of the endoscope without dilation. At 24 months, new brownish lesions at 31–32 and 36 cm with early cancer features were treated by endoscopic mucosal resection, and pathology again confirmed SBSCC (M2) ( Fig. 5 , Video 1 ). No invasive carcinoma or metastasis was detected throughout the follow-up.

Endoscopy_UCTN_Code_CCL_1AB_2AC_3AB
